# Artificial Intelligence in Ophthalmology: Acceptance, Clinical Integration, and Educational Needs in Switzerland

**DOI:** 10.3390/jcm14176307

**Published:** 2025-09-06

**Authors:** Christoph Tappeiner

**Affiliations:** 1 Department of Ophthalmology, Pallas Kliniken, 4600 Olten, Switzerland; christoph.tappeiner@pallas-kliniken.ch; 2 Medical Faculty, University of Bern, 3008 Bern, Switzerland; 3 Department of Ophthalmology, University Hospital Essen, University Duisburg-Essen, 45147 Essen, Germany; 4 Private Hochschule Wirtschaft PHW, 3014 Bern, Switzerland

**Keywords:** artificial intelligence (AI), ophthalmology, digital affinity, barriers, training, Switzerland

## Abstract

**Background:** Artificial intelligence (AI) can improve efficiency, documentation, and diagnostic quality in ophthalmology. This study examined clinical AI adoption, institutional readiness, perceived utility, trust, ethical concerns, and educational needs among Swiss ophthalmologists and residents. **Methods:** In May 2025, an anonymous online survey was distributed to board-certified ophthalmologists and residents across Switzerland. The structured questionnaire addressed clinical AI use, institutional infrastructure, perceptions of diagnostic utility, trust, ethical–legal concerns, and educational preparedness. Responses were recorded on five-point Likert scales. **Results:** Of 106 respondents (mean age 42.4 ± 11.4 years, 48.1% female), 20.8% reported current clinical AI use. Willingness to use AI exceeded 65% across all 10 diagnostic scenarios, but active use remained ≤12.1%. Institutional readiness was low: 6.6% reported AI-related guidelines, 26.4% had access to an institutional AI contact person, and 28.3% received supervisor support (more often among residents). While 80% agreed that AI can support diagnostics, only 12.1% trusted AI recommendations as much as those from colleagues; 87.9% critically reviewed the results, and 93.9% endorsed the use of AI in an assistive but not independently decision-making role. Ethical–legal concerns included unresolved liability (74.8%), informed consent (66.7%), and data protection adequacy (49.5%). Structured AI education was supported by 77.8%, yet only 15.1% felt prepared, and two-thirds (66.7%) indicated they would use AI more with better training. **Conclusions:** Ophthalmologists and residents in Switzerland express strong interest in the clinical use of AI and recognize its diagnostic potential. Major barriers include insufficient institutional structures, lack of regulatory clarity, and inadequate educational preparation. Addressing these deficits will be essential for responsible AI integration into ophthalmologic practice.

## 1. Introduction

Artificial intelligence (AI) is increasingly integrated into clinical practice across medical disciplines. By processing large datasets, identifying complex patterns, and providing decision support, AI can enhance diagnostic accuracy, improve workflow efficiency, and increase healthcare accessibility [1]. Ophthalmology, as a specialty relying heavily on imaging, is particularly well-positioned to benefit from these advances. Deep-learning algorithms have demonstrated high performance in classifying retinal diseases using fundus photography and optical coherence tomography (OCT) [2,3,4]. Automated tools for detecting diabetic retinopathy, age-related macular degeneration, and glaucoma are in clinical use or pending regulatory approval in several countries [5].

Table 1 provides an overview of the current landscape of AI integration in ophthalmology, summarizing established and emerging clinical applications across the main subspecialties. These include AI-based tools for screening and diagnosis of retinal diseases such as diabetic retinopathy and age-related macular degeneration, automated algorithms for glaucoma detection and progression monitoring, anterior segment imaging analysis, including keratoconus detection, and applications in ocular surface disease screening. Beyond clinical diagnostics and disease management, AI tools are increasingly supporting administrative workflows, surgical planning, and medical education and training, underscoring the rapidly expanding role of AI across both clinical and non-clinical domains in ophthalmology.

Despite these technological advances, the integration of AI into routine ophthalmic practice remains limited. International surveys have identified persistent barriers, including clinician skepticism, ethical and legal uncertainties, and insufficient training [33,34,35].

In Switzerland, despite a well-developed healthcare system and strong interest in innovation, no national guidelines from the Swiss Society of Ophthalmology (SOG) or the Swiss Institute for Postgraduate and Further Education in Medicine (SIWF) currently outline the integration, evaluation, or ethical governance of AI in clinical ophthalmology. The Swiss Medical Association (FMH) has published initial position statements and reports on the use of AI in medicine [36,37], but specific guidance for ophthalmology is not yet available. Educational gaps further hinder adoption. The Swiss residency training program in ophthalmology, as defined by the SIWF/FMH/SOG framework, contains no AI-specific content [38]. The demand for structured AI education is reflected in global reports [39,40], yet systematic implementation in Switzerland is lacking.

This study presents an assessment of AI use, perceptions, and educational needs among ophthalmologists and residents in Switzerland. The analysis covers current clinical applications, perceived utility and trust, ethical concerns, and self-assessed readiness, with attention to subgroup differences (e.g., age, gender, career stage), institutional contexts, and specific educational demands. The findings aim to support the development of guidelines, the creation of AI-related training programs, and the responsible clinical implementation of AI in ophthalmology.

## 2. Methods

### 2.1. Study Design and Setting

A cross-sectional, anonymous, web-based survey was conducted, targeting board-certified ophthalmologists and residents in ophthalmology training across Switzerland. The survey was conducted in May 2025 using the findmind.ch platform, which complies with Swiss data protection standards. Participation was entirely voluntary.

### 2.2. Participant Recruitment

Participants were recruited through multiple channels to reach a broad cross-section of ophthalmologists in Switzerland. The invitation link to the final online survey was distributed via the Young Swiss Ophthalmologists (YSO) WhatsApp group, which includes residents and young specialists within the Swiss Society of Ophthalmology (SOG). In addition, clinical directors of ophthalmology departments across Switzerland were contacted by email and asked to forward the invitation to all ophthalmologists in their teams. Further, practicing ophthalmologists in private settings were individually invited via email, using available professional networks, to ensure broad geographical and institutional coverage. This approach aimed to minimize bias regarding age, level of training, and region. We estimate that approximately one quarter of ophthalmologists in Switzerland (i.e., about 300 ophthalmologists) were directly reached by the survey invitation. Inclusion criteria were a current activity in ophthalmology within Switzerland, ability to read one of the survey languages (German, French, Italian, or English), and informed consent to participate in the anonymous survey.

### 2.3. Sample Size Determination

The target population consisted of all ophthalmologists practicing in Switzerland (*N* = 1.194, according to the FMH physician statistics, 2024) [41]. The required sample size was calculated using a 95% confidence level (*z* = 1.96), a 10% margin of error (*e* = 0.10), and a conservative expected proportion (*p* = 0.5), applying the finite population correction formula. This resulted in a minimum required sample size of 89 completed questionnaires to obtain statistically reliable estimates.

### 2.4. Questionnaire Development

The survey was developed following a literature review and incorporated elements from established technology acceptance frameworks [42], ensuring that all relevant aspects of AI use and acceptance among ophthalmologists were adequately covered. The questionnaire consisted of different thematic blocks covering demographics (including gender, age, professional experience, canton of workplace, and institutional setting), digital interest and experience, private AI use, clinical AI applications, diagnostic scenarios, perceptions of AI utility and trust, ethical and legal dimensions, institutional readiness, and educational preparedness.

All thematic blocks used structured items rated on a five-point Likert scale (1 = strongly disagree/never/very low; 5 = strongly agree/daily/very high), depending on the question type. Some sections additionally assessed both active use and willingness to use (e.g., diagnostic scenarios). The complete questionnaire, including all survey items and response options, is available as Supplementary Material (File S1). Prior to rollout, the questionnaire was pilot-tested with three ophthalmologists from different institutional settings to evaluate content validity, clarity, and completion time; minor revisions were made to wording and layout based on feedback.

### 2.5. Data Collection and Statistical Analysis

Responses were collected over a 3-week period (1–21 May 2025). Data were downloaded in CSV format, screened for missing data, duplicates, and implausible values. Statistical analysis was performed using jamovi version 2.7.2 [43] and the vijPlots module [44]. Descriptive statistics were calculated for all variables. Continuous variables were reported as means ± standard deviations; categorical variables as frequencies and percentages. Group comparisons (e.g., residents vs. specialists, male vs. female, high vs. low digital affinity) were performed using Mann–Whitney U tests for continuous variables and chi-square tests for categorical variables, with Holm correction for multiple comparisons. Multiple linear regression was used to identify predictors of digital interest and experience, with gender, age, training status, and institutional setting as covariates. Simple linear regression was used to examine associations between age and the digital interest/experience score. A *p*-value of <0.05 was considered statistically significant.

## 3. Results

### 3.1. Sample Characteristics

Of the 106 valid responses, 51 were female (48.1%) and 55 were male (51.9%). The mean age was 42.4 years (SD = 11.4, range: 22–70). Mean professional experience was 14.0 ± 10.5 years (range: 1–41). The sample included both residents (24.5%) and board-certified ophthalmologists (75.5%). Participants represented a diverse set of institutional backgrounds and geographic regions. Geographically, physicians were distributed across Switzerland. The largest proportion practiced in the Canton of Zurich (20.8%), followed by Bern (17.9%), Ticino (10.4%), Solothurn (9.4%), Aargau (8.5%), and Basel-City (7.5%), with the remainder located in other cantons. Most respondents reported working in private practices (47.2%), followed by private hospitals (13.2%) and university hospitals (12.3%).

### 3.2. Digital Interest and Experience (IE)

To assess digital competence, as well as openness toward digital technologies and artificial intelligence, participants rated eight statements on a five-point scale (1 = very low, 5 = very high). These covered general software skills (IE1: 3.14 ± 1.09), basic knowledge of AI functionality (IE2: 2.96 ± 1.09), private experience with AI applications (IE3: 2.98 ± 1.19), professional AI experience (IE4: 2.36 ± 1.24), knowledge of digital data protection (IE5: 2.94 ± 1.18), enjoyment of experimenting with new software (IE6: 3.46 ± 1.45), interest in digital health technologies (IE7: 3.88 ± 1.23), and interest in the use of AI (IE8: 4.01 ± 1.18). Significant subgroup differences were observed: Men scored higher than women in five of eight items: general software skills (IE1: *p* = 0.013), private experience with AI applications (IE3: *p* = 0.013), enjoyment of experimenting with new software (IE6: *p* < 0.001), interest in digital health technologies (IE7: *p* = 0.002), and interest in AI use (IE8: *p* = 0.002). Assistants scored higher than specialists in IE2 (*p* = 0.032) and IE6 (*p* = 0.029). Age-related differences were significant for the overall mean scale score (*p* = 0.048), with ≤40 years showing higher mean values.

**Figure 1:**
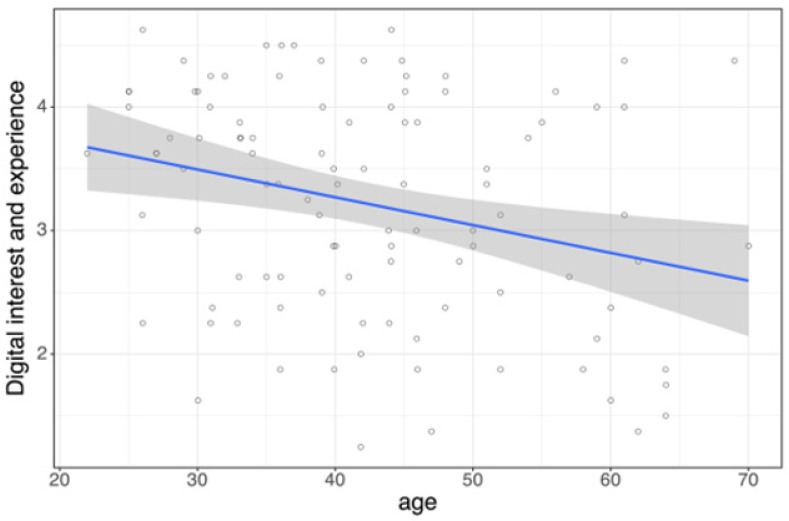
Age as a predictor of digital interest and experience is shown as a linear regression plot with 95% confidence interval (*p* = 0.004, *R*^2^ = 0.0799).

Multiple linear regression analysis revealed that gender (*β* = −0.707, *p* < 0.001) and age (*β* = −0.027, *p* < 0.001) were significant predictors of digital interest and experience (*R*^2^ = 0.236), whereas workplace type showed no effect (*p* = 0.781). Simple linear regression confirmed a negative association between age and digital interest and experience (*β* = −0.0225, *p* = 0.004, *R*^2^ = 0.0799; Figure 1).

### 3.3. Private Use (PU) of AI-Based Technologies

In the context of private use of AI-based technologies, the highest reported frequency (daily or at multiple times per week) was for text correction or improvement (PU1: 47.5%), followed by translation tools (PU6: 46.6%), text generation (PU2: 26.2%), and navigation/route planning (PU8: 24.3%). Less common were speech-to-text transcription (PU5: 19.4%) and voice assistants (PU7: 17.4%). Visual AI applications were rare: image generation/editing (PU3: 5.9%) and video generation/editing (PU4: 2.0%). “Never” responses peaked for video generation/editing (81.6%), image generation/editing (47.6%), and speech-to-text transcription (43.7%). Significant subgroup effects were found. Men used voice assistants more often than women (PU7: *p* = 0.021). Residents reported more frequent text generation than specialists (*p* < 0.001). No significant age differences were found.

### 3.4. Use of AI at the Workplace (WP)

**Figure 2:**
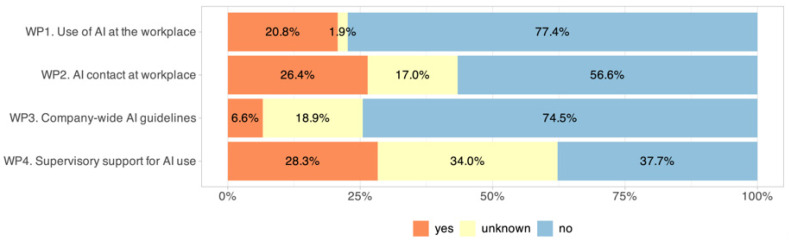
Institutional support for AI adoption, showing the proportion of respondents reporting the availability of institutional structures facilitating clinical implementation, such as access to AI guidelines, designated AI contact persons, and supervisor encouragement.

AI implementation in daily clinical routines remained limited. Only 20.8% of respondents indicated that they use AI-based tools provided in their institution at least occasionally (Figure 2, WP1). In contrast, institutional preparedness was insufficient. Access to a dedicated AI expert was available to just 26.4% of respondents (WP2). Only 6.6% worked in an environment with official AI guidelines (WP3), and fewer than one-third (28.3%) reported any support or initiative from senior leadership regarding AI integration (WP4). Residents reported more frequent support from supervisors (57.7% vs. 18.8% in specialists, *p* < 0.001). No gender or age effects were found.

### 3.5. Most Common AI Tools in Clinical Practice (CP)

**Figure 3:**
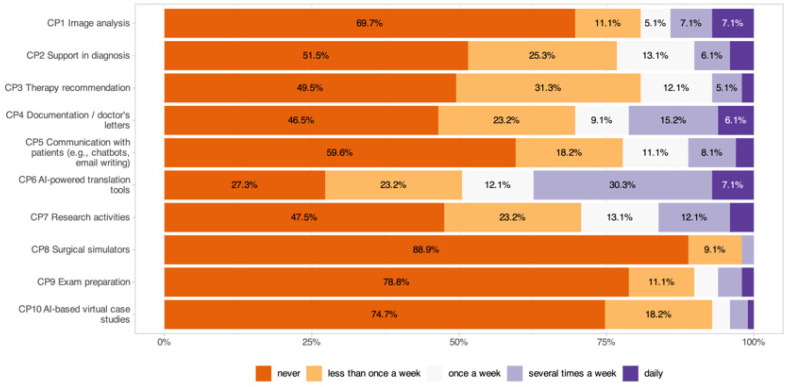
Frequency of AI tool usage in clinical practice across different application areas.

The frequency of AI tool usage (1 = never, 5 = daily; Figure 3) showed that translation tools were the most frequently used application, with 49.5% of respondents using them at least once per week (CP6: 2.67 ± 1.35), followed by documentation support (30.4%, CP4: 2.11 ± 1.31), scientific work (29.2%, CP7: 2.02 ± 1.21), and diagnostic decision support (23.2%, CP2: 1.86 ± 1.12). Therapy recommendations (CP3: 1.79 ± 0.98), patient communication tools (CP5: 1.77 ± 1.12), and image analysis (19.3%, CP1: 1.71 ± 1.26) were used less frequently. The least commonly used applications were virtual case studies (>70% never, CP10: 1.37 ± 0.78), exam preparation (>70% never, CP9: 1.39 ± 0.90), and surgical simulators (>70% never, CP8: 1.13 ± 0.40).

Significant subgroup effects were identified, with younger participants using exam preparation tools more often (*p* = 0.004) and residents using AI tools for scientific work (CP7: *p* < 0.001) and exam preparation (CP9: *p* < 0.001) more frequently than specialists. High digital affinity was associated with greater use of documentation (CP4: *p* = 0.010), patient communication (CP5: *p* = 0.010), and surgical simulators (CP8: *p* = 0.004).

### 3.6. Diagnostic (D) Applications

**Figure 4:**
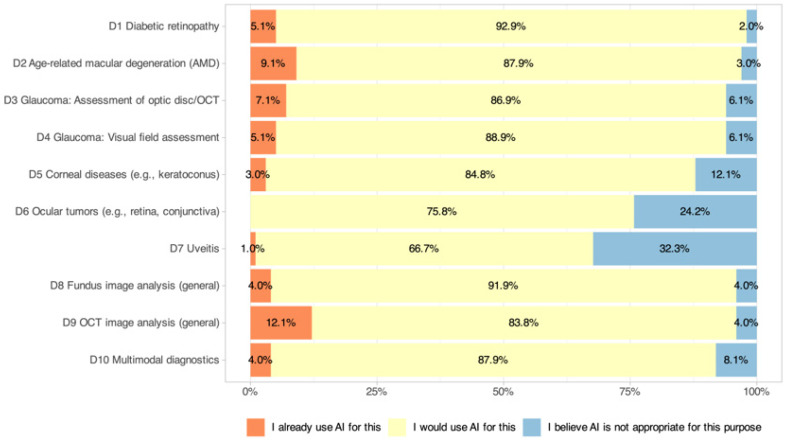
Current use and willingness to adopt AI-based diagnostic applications in clinical practice among Swiss ophthalmologists. The figure displays responses across 10 diagnostic scenarios.

The current usage or willingness to introduce diagnostic AI applications into clinical practice is shown in Figure 4. Across all 10 scenarios, willingness to use AI exceeded 65% (range: 66.7–92.9%), but active use remained low (range: 0–12.1%). OCT image analysis (D9: 12.1% active, 83.8% willingness) had the highest active use, followed by AI analysis of AMD (D2: 9.1% active, 87.9% willingness), OCT optic nerve head for glaucoma (D3: 7.1% active, 86.9% willingness), and visual fields for glaucoma (D4: 5.1% active, 88.9% willingness). Diabetic retinopathy (D1: 5.1% active, 92.9% willingness) also showed high willingness despite low active use. Moderate levels were seen for corneal diseases (D5: 3.0% active, 84.8% willingness), fundus image analysis (D8: 4.0% active, 91.9% willingness), and multimodal diagnostics (D10: 4.0% active, 87.9% willingness). The least common active uses were for ocular tumors (D6: 0% active, 75.8% willingness) and uveitis (D7: 1.0% active, 66.7% willingness). Significant subgroup effects were identified, with residents reporting more active use for diabetic retinopathy than specialists (D1: *p* = 0.005) and high digital affinity correlating with higher active use of AI in fundus image analysis (D8: *p* = 0.002) and multimodal diagnostics (D10: *p* = 0.004). No significant gender or age effects were found.

### 3.7. Potential Applications of AI in Ophthalmology

**Figure 5:**
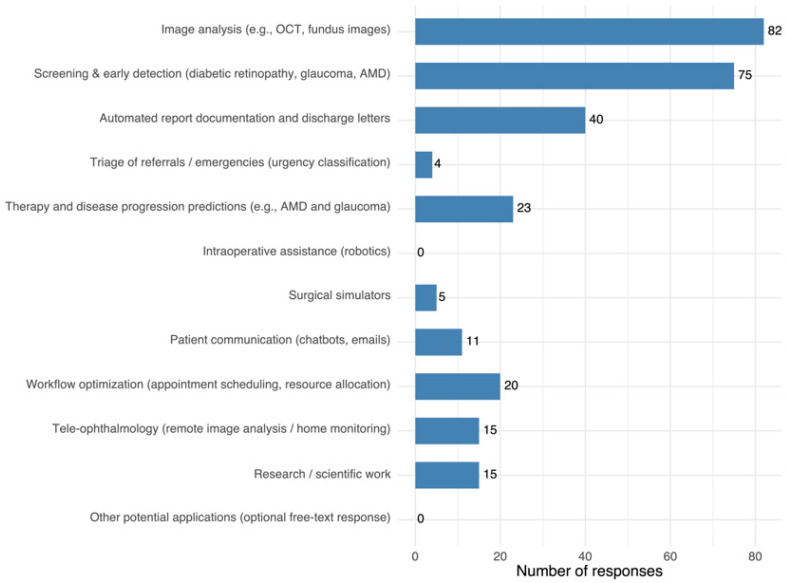
Most frequently cited applications with the highest perceived potential of AI use in ophthalmology. Each participant could select up to three options.

When asked about areas where AI could be most useful, respondents most frequently cited image analysis (e.g., OCT, fundus photography) and screening/early detection (e.g., diabetic retinopathy, glaucoma, AMD), followed by automated report generation. Other applications, such as therapy/prognosis, workflow optimization, teleophthalmology, research, and patient communication, were mentioned less often, with intraoperative assistance not selected at all. Full distribution of responses is shown in Figure 5.

### 3.8. Main Barriers to AI Adoption

**Figure 6:**
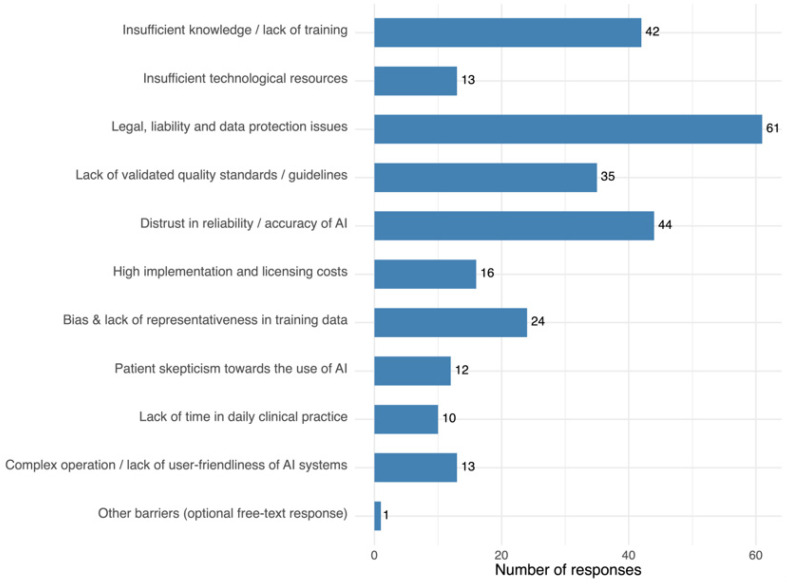
Most frequently cited barriers to AI implementation. Each participant could select up to three options.

The most frequently reported barriers were legal, liability, and data protection concerns, mistrust in reliability/accuracy, and insufficient knowledge/training. Lack of validated quality standards/guidelines and bias in training data were also noted, alongside cost, usability, and resource issues. Only one participant provided a free-text response for the barriers item (“no interest; unnecessary; an additional burden for physicians; IT should be fully responsible for this”). Complete results are presented in Figure 6.

### 3.9. Perceptions of AI Utility, Accuracy, and Trust (UAT)

**Figure 7:**
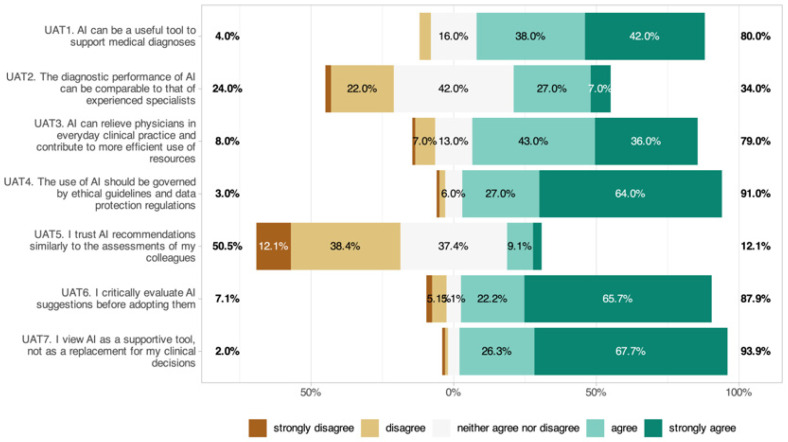
Perceptions of AI utility, accuracy, and trust among ophthalmologists.

The perception of AI utility, accuracy, and trust is shown in Figure 7. A majority (80%) agreed AI could support diagnostics (UAT1: 4.18 ± 0.85), but only 34% believed AI could match the accuracy of experienced ophthalmologists (UAT2: 3.15 ± 0.91). Seventy-nine percent believed AI could improve workflow efficiency (UAT3: 4.06 ± 0.93). Ninety-one percent endorsed ethical guidelines and data protection (UAT4: 4.51 ± 0.79). Only 12.1% trusted AI as much as colleagues (UAT5: 2.53 ± 0.93), 87.9% critically reviewed outputs (UAT6: 4.44 ± 0.95), and 93.9% saw AI as a subordinate tool (UAT7: 4.59 ± 0.71). Significant subgroup effects were identified, with men expressing more trust in AI recommendations (UAT1: *p* = 0.015) and higher digital affinity being linked to greater trust (UAT1: *p* = 0.003). No significant age or career-level effects were found.

### 3.10. Ethical and Legal Dimensions (EL)

**Figure 8:**
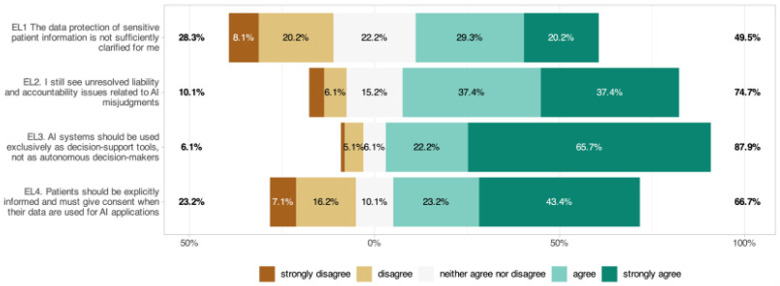
Respondents’ perspectives on ethical and regulatory considerations in AI integration within ophthalmology.

While 28.3% of respondents felt that data protection was adequately clarified when using AI, 49.5% indicated that it was not (Figure 8, EL1: 3.33 ± 1.24). Also, 74.8% cited unresolved liability issues (EL2: 3.98 ± 1.07); 87.9% wanted AI only as a decision-support tool (EL3: 4.46 ± 0.90); and 66.7% required explicit patient consent (EL4: 3.80 ± 1.34). Significant subgroup effects were identified, with women agreeing more strongly with the need for patient consent (EL4: *p* = 0.010) and specialists agreeing more often than residents that AI systems should be used exclusively as decision-support tools (EL3: *p* = 0.015). No significant age or digital affinity effects were found.

### 3.11. Educational Gaps and Learning Needs (LN)

When asked about training, 77.8% supported integrating structured AI education into ophthalmology curricula (LN4: 4.22 ± 0.90), while only 15.1% had meaningful exposure in training (LN6: 2.24 ± 1.10). Two-thirds (66.7%) would use AI more with better training (LN5: 3.88 ± 1.13). Significant subgroup effects were identified, with participants with high digital affinity more often believing that AI will reduce workload (LN3: *p* = 0.014). No significant gender, age, or training-level effects were found.

### 3.12. Analysis of Free-Text Responses

In the optional open-ended question, participants were invited to share additional perspectives and suggestions regarding the use of AI in ophthalmology. In total, six evaluable responses were submitted. The exploratory thematic analysis revealed several recurring themes:

Skepticism and limited trust in current AI solutions: Multiple participants expressed concerns regarding the clinical reliability of current AI outputs. For example, one participant reported that “the diagnoses provided by AI were very far from real clinical diagnoses,” while another stated that they currently have no trust in AI due to prior experiences with incorrect diagnostic suggestions.

Need for medical validation and clear assignment of responsibilities: One respondent emphasized that AI-generated suggestions should always be validated by physicians, and that IT-related aspects should remain within the scope of IT departments to avoid additional burdens for clinicians.

Regulatory guidance and integration into existing systems: Some participants recommended that professional societies (e.g., FMH, SOG) publish trusted recommendations for AI tools, including specific guidance for different applications. Concerns were also raised about outsourcing diagnostic competencies to non-medical providers and the need for protective regulations at the national level.

Implementation barriers and infrastructural challenges: Another key theme was the lack of robust technical infrastructure and inefficiencies in existing clinical IT systems. Respondents noted that, at present, improving current workflows yields more efficiency gains than AI solutions, though they acknowledged potential future benefits in screening and documentation.

Desire for transparency and comparability of AI tools: One participant highlighted the importance of transparency and the ability to compare different AI providers, noting that some tools are more advanced and better integrated than others.

## 4. Discussion

This survey offers an up-to-date view of how Swiss ophthalmologists perceive and use AI. Across domains, openness was high, but clinical integration remained modest: only 20.8% reported any institutional AI use, formal AI guidelines were rare, and access to dedicated AI expertise was limited. The resulting intention–implementation gap was most visible in diagnostic scenarios, where willingness exceeded 65% for all 10 indications (range: 66.7–92.9%), but active use remained ≤12.1% for all scenarios. Similar adoption frictions, driven more by structural than attitudinal barriers, have been described across medical fields [34].

Digital interest and experience were shaped by demographic factors already noted in other medical cohorts. Younger age and male gender were associated with higher digital affinity, and residents reported higher scores on selected items, consistent with broader evidence that younger physicians are readier to engage with emerging technologies [45,46,47]. Still, both residents and specialists endorsed AI’s potential for efficiency gains, and high digital affinity correlated with a stronger belief that AI may reduce workload, underscoring the role of attitudes in shaping expectations.

The pattern of clinical interest centered on standardized, image-based tasks, e.g., OCT analysis, DR screening, and AMD assessment, where high-performing models already exist [2,3,4,5]. That active use still lagged behind willingness even in these domains suggests that validation alone is insufficient: integration into workflows, governance, and training must evolve in parallel. By contrast, more complex and less standardized indications (e.g., ocular tumors, uveitis) elicited lower willingness, revealing clinicians’ preference for human oversight when uncertainty is high.

Perceptions of utility were favorable; 80% agreed AI can support diagnostics, and 79% saw potential efficiency gains, yet trust remained circumscribed. Only 12.1% trusted AI as much as colleagues; 87.9% reported critically reviewing outputs; and 93.9% endorsed AI in a subordinate role. This posture mirrors the “human-in-the-loop” paradigm, emphasizing clinician accountability and contextual judgment [48]. Men expressed more trust in AI recommendations, and higher digital affinity was linked to greater trust.

Ethical–legal concerns were prominent. Three-quarters cited unresolved liability, only 28.3% felt data protection is adequately clarified, 66.7% required explicit patient consent, and 87.9% wanted AI restricted to decision support, with specialists more often endorsing the support-only view. These positions align with international calls for transparent, accountable, and controllable AI in health systems [49], and with analyses underscoring the centrality of liability clarity for responsible deployment [50]. They also resonate with Swiss discourse: FMH has emphasized ethical use, data protection, and clear responsibilities [36,37], while SIWF/FMH currently lacks AI-specific requirements in the ophthalmology curriculum, mirroring our finding of strong demand for structured training given the minimal prior exposure to AI in clinical practice [38].

The educational signal was consistent: 77.8% supported integrating structured AI education, only 15.1% reported meaningful AI exposure, and two-thirds would use AI more with better training. International experiences suggest that targeted curricula can increase acceptance and skills [40,51]. Importantly, our subgroup result that high digital affinity relates to perceived workload relief suggests that curricula should combine foundational competencies with realistic, workflow-aware use cases to convert interest into safe adoption. Findings from other Swiss research reinforce these conclusions. In a study, presented by Swiss ophthalmologists at the ARVO 2025 meeting, a single 90 min educational session on medical AI led to an increase in self-reported knowledge [52]. However, no relevant changes were observed in acceptance of AI or willingness to implement it in clinical practice. The authors concluded that brief educational interventions may enhance knowledge but are unlikely to meaningfully influence adoption attitudes on their own. They emphasized the need for sustained, curriculum-integrated training and concurrent institutional support to foster broader AI uptake in ophthalmology [52].

Compared with international surveys, Swiss ophthalmologists display similarly high openness to AI and strong expectations for decision–support benefits, consistent with multinational data in which 88.1% of respondents reported openness to using AI as a clinical assistance tool [53]. The demographic gradients we observed, i.e., greater digital affinity among younger physicians and among men, mirror patterns reported in other medical cohorts [45,46,47,54,55]. The preference for clinician-supervised, “human-in-the-loop” use aligns with international perspectives on accountability in AI-supported care [56]. Compared to other countries, Switzerland still lags behind in the structured integration of AI content into medical education and training. While some countries have already implemented standardized curricula and practice-oriented training programs [57,58], Switzerland currently lacks mandatory, curriculum-based modules that address technical foundations, legal frameworks, and practical application scenarios. This gap makes it more difficult to build consistent competencies among ophthalmologists and to ensure the safe and efficient integration of AI into clinical workflows. Moreover, the lack of uniform, standardized survey instruments across countries makes direct comparisons challenging. Regular monitoring of global developments and structured international exchanges would be highly beneficial to identify trends, share best practices, and facilitate harmonized strategies for AI adoption in ophthalmology.

Several strengths enhance interpretability of the current survey: nationwide reach across practice settings, inclusion of both trainees and specialists, and a pretested, structured instrument spanning digital affinity, clinical applications, perceptions, ethics/legal, and education. Limitations include the cross-sectional design, reliance on self-report (susceptible to social desirability) [59], and potential selection of digitally interested participants. A self-selection bias cannot be excluded, as participation was voluntary and ophthalmologists with a greater interest in digital topics or AI may have been more likely to respond, while more skeptical or less digitally affine physicians may be underrepresented. Only about one-quarter of ophthalmologists in Switzerland (approximately 300 individuals) were directly reached by the survey invitation; however, 106 completed the questionnaire, corresponding to a response rate of approximately 35%. While this exceeds the calculated minimum sample size and supports the representativeness of the results, a non-response bias cannot be fully ruled out. Subgroup findings were exploratory and, despite meeting the a priori sample size, may be underpowered for some effects. Generalizability beyond Switzerland is uncertain, and rapid technological and regulatory changes limit temporal validity [1].

Although AI shows clear promise for screening, diagnosis, and workflow support in ophthalmology, several constraints still limit its reliable and equitable deployment in ophthalmology. Technically, data heterogeneity, limited metadata standards, and weak interoperability with electronic records impede seamless integration into clinical workflows [10,15]. Model performance can degrade when applied to data that differ from the datasets used for development, and hidden biases, such as racial, geographic, or socioeconomic imbalances, may limit generalizability and risk reinforcing healthcare inequities if systems are not continuously monitored and audited [23,60,61,62]. The “black-box” nature of many deep-learning models further limits interpretability and clinical trust, underscoring the need for transparent and explainable algorithms [63,64]. Ethical and legal uncertainties remain significant, including unresolved liability, informed consent, and privacy issues, as well as the absence of standardized validation and governance frameworks [34,49,50,65]. Resource-related barriers, such as the need for large, high-quality annotated datasets, secure infrastructure, and robust cybersecurity measures, further hinder practical implementation, particularly outside tertiary or academic centers [12,23,33]. Going forward, carefully designed studies embedded in real clinical workflows, along with continuous monitoring and transparent reporting of errors, are needed to ensure safe and evidence-based adoption of AI in practice [66]. In addition, incorporating AI education into training programs, supported by clear guidelines and institutional policies, will be key to clarifying responsibilities and ensuring informed patient consent [36,37].

Successful integration of AI in ophthalmology will require a coordinated and multidimensional approach. Rather than reflecting fundamental resistance, the barriers identified in this study highlight structural, regulatory, and organizational gaps that must be addressed [34,36,37,65]. National societies, such as the Swiss Society of Ophthalmology (SOG), Swiss Medical Association (FMH), and the Swiss Institute for Postgraduate and Further Education in Medicine (SIWF), should take the lead in developing evidence-based guidelines for the adoption of AI. These guidelines need to define validation and quality assurance requirements, ensure transparency and explainability, address liability and data protection, and provide consistent frameworks for clinical use across practice settings. AI education should be systematically integrated into residency curricula and continuing professional development, with structured, hands-on training modules embedded in clinical workflows to build competence and confidence among ophthalmologists [57,58,67]. Institutional governance must advance in parallel by establishing clear processes for evaluating, approving, and monitoring AI applications, ensuring interoperability with existing clinical information systems, and appointing designated AI leads or interdisciplinary working groups to support safe and effective implementation. Transparent communication and structured risk disclosure are equally critical, as trust in AI depends on the perceived explainability and accountability of these systems [64]. In addition, pilot implementation multicenter studies can provide essential evidence on feasibility, equity, and cost-effectiveness in real-world settings. By addressing these areas in a coordinated and evidence-driven manner, Switzerland can transition from high willingness but low implementation toward the safe, effective, and ethically responsible integration of AI in ophthalmology.

In the Swiss context, three plausible development scenarios can be envisioned for the integration of AI in ophthalmology. In an optimistic scenario, AI-supported systems would be rapidly implemented across all levels of care, with automated image analysis, intelligent screening, and assisted therapeutic decision-making becoming part of routine workflows. Switzerland could establish itself as one of the leading countries in the ethical and quality-assured integration of AI, provided that stable regulatory, technical, and institutional frameworks are in place. This would align with the WHO’s principles for the responsible use of AI, which emphasize human oversight, transparency, accountability, and robust data protection [49]. A probably more realistic scenario would likely involve selective adoption in standardized, imaging-driven areas such as retina and glaucoma diagnostics, where validated systems already exist. Implementation would proceed stepwise, depending on infrastructure, institutional support, and the digital literacy of clinicians. In a pessimistic scenario, unclear regulations, limited financial incentives, and cultural resistance could slow or even prevent wider adoption of AI, leading to minimal clinical integration and an increasing gap compared with international standards. To move towards more favorable outcomes, professional societies, training institutions, hospital administrations, and policymakers must work together to ensure that AI is implemented in a responsible, transparent way that delivers clear and measurable benefits for patient care.

## 5. Conclusions

Overall, Swiss ophthalmologists show high willingness but low active use of AI, particularly outside image-based diagnostics. The principal obstacles include limited institutional readiness, liability and data protection uncertainty, circumscribed trust, and scarce training, all of which are addressable. Progress will depend on coordinated action: (i) specialty-specific governance and guidance that clarify accountability and data use [36,37,49]; (ii) integration of AI competencies into SIWF/FMH training pathways with hands-on, workflow-relevant content [38,40,51]; and (iii) institutional support for implementation and evaluation. Given ophthalmology’s imaging-centric workflows and the maturity of several validated tools [2,3,4,5], the specialty is well-positioned to benefit if adoption is evidence-based, ethically governed, and aligned with clinical priorities.

Taken together, these findings indicate that AI in ophthalmology is at a pivotal point. There is broad openness and recognition of its potential to enhance diagnostics, streamline workflows, and improve efficiency, yet integration into daily clinical practice remains limited and still focused mainly on image-based applications. This highlights the importance of establishing clear regulatory frameworks, offering structured and hands-on training, and strengthening institutional support to ensure safe and responsible adoption. With its imaging-centric workflows and standardized diagnostic pathways, ophthalmology is particularly well-positioned to benefit from AI. Moving forward, coordinated efforts by clinicians, professional societies, training institutions, and policymakers will be essential to turn AI’s potential into measurable improvements in clinical care, ensuring that its implementation is fair, transparent, and consistent with the highest standards of patient safety and quality.

## Figures and Tables

**Table 1:** Clinical applications of artificial intelligence in ophthalmology with representative examples.

Application Category	Examples	References
Retinal Disease Diagnosis and Management	Diabetic retinopathy: screening/classification; AMD: detection, grading, progression monitoring; prediction of treatment need and response; Retinal vein occlusion: detection on OCT and fundus images; Retinopathy of prematurity: detection and severity grading	[6,7,8,9,10,11,12,13]
Glaucoma Care	Automated glaucoma detection from fundus/OCT images, visual field progression analysis	[7,12,14,15,16,17]
Anterior Segment Diseases	Keratoconus: detection/classification from corneal topography, evaluation of progression; Infectious keratitis: automated diagnosis from slit-lamp images, treatment response evaluation; Cataract: automated grading from slit-lamp images; Dry eye: meibomian gland analysis; Pterygium: detection and progression analysis on anterior segment images; Conjunctival tumors: AI-based detection and risk stratification	[10,14,18,19,20,21]
Ophthalmic Oncology	Choroidal melanoma: detection/classification from fundus images; Retinoblastoma: automated screening; Ocular surface tumors: detection and AI-based risk stratification	[13,14,20,22]
Multimodal AI Systems	Integrated imaging–clinical–genomic models: comprehensive diagnosis and prognostic assessment	[14,15,23]
Surgical Assistance and Planning	Cataract/refractive/retinal surgery: AI-based planning and intraoperative guidance; Postoperative complication prediction: automated risk models	[14,15,23,24]
Image Enhancement and Segmentation	Fundus/OCT/anterior segment: AI-based image enhancement, automated segmentation of retinal layers/lesions, detection of intraretinal and subretinal fluid, choroidal neovascularization, etc.	[13,14,20,25]
Predictive Analytics and Risk Stratification	Visual outcome prediction: AI-based models; High-risk patient identification: risk scoring; Personalized management: treatment response prediction; AI models predicting surgical outcomes in complex surgical cases	[12,23,26,27]
Public Health and Population Screening	Automated diabetic retinopathy/glaucoma/cataract/myopia screening; Risk prediction; Telemedicine screening; AI-driven mobile apps for early detection in underserved regions	[6,8,10,28,29,30]
Education and Training	Surgical simulation platforms: AI-assisted virtual reality simulators for eye surgeries; Automated skill assessment; Intraoperative feedback and assistance; Adaptive learning modules; Clinical decision support; Use of generative AI for interactive case-based learning modules	[8,14,31]
Administrative and Workflow Optimization	Scheduling; Triage; Automated documentation; Resource allocation; Chatbot-based patient communication; Virtual assistants; Patient education; AI-assisted clinical coding and billing	[8,10,19,31,32]

## Data Availability

The raw data supporting the conclusions of this article are available from the authors upon reasonable request.

## Supplementary Materials

The following supporting information can be downloaded at: https://www.mdpi.com/article/10.3390/jcm14176307/s1, Survey questionnaire (English version).
