# Supplementary material for: Artificial Intelligence in Ophthalmology: Acceptance, Clinical Integration, and Educational Needs in Switzerland
Source: J Clin Med. 2025 Sep 6;14(17):6307. doi: 10.3390/jcm14176307 (PMC12429519; doi:10.3390/jcm14176307)
Supplement: Supplementary file 1 [file jcm-14-06307-s001.zip › Supplementary File S1.pdf]

Artificial Intelligence in Ophthalmology
A Survey on the Acceptance and Use of AI Among Swiss Ophthalmologists

Sociodemographic and professional background

Gender
- ◯ male
- ◯ female
- ◯ diverse

Age

____

Canton
- ◯ (currently abroad)
- ◯ Aargau
- ◯ Appenzell Ausserrhoden
- ◯ Appenzell Innerrhoden
- ◯ Basel-Landschaft
- ◯ Basel-Stadt
- ◯ Bern
- ◯ Freiburg
- ◯ Geneva
- ◯ Glarus
- ◯ Graubünden
- ◯ Jura
- ◯ Lucerne
- ◯ Neuchâtel
- ◯ Nidwalden
- ◯ Obwalden
- ◯ Schaffhausen
- ◯ Schwyz
- ◯ Solothurn
- ◯ St. Gallen
- ◯ Ticino
- ◯ Thurgau
- ◯ Uri
- ◯ Vaud
- ◯ Valais
- ◯ Zug
- ◯ Zurich

Level of education
- ◯ I am a resident
- ◯ I am a board-certified specialist

Professional experience
How many years have you been working in ophthalmology

____

Place of work

☐ private practice
☐ Cantonal Hospital
☐ private hospital
☐ University Hospital
☐ Non-clinical research laboratory
☐ other

AI implementation in the work environment / institution

	Yes	no	I do not know
(WP1) Is AI currently in use at your workplace (e.g. AI for OCT analysis or letter generation)?	◯	◯	◯
(WP2) Is there a designated AI contact person at your employer (IT and/or research)?	◯	◯	◯
(WP3) Has your employer published a formal AI policy?	◯	◯	◯
(WP4) Do your superiors explicitly support the use of AI?	◯	◯	◯

Affinity for and interest in technology

Interest and experience with IT / software (general)
Please rate each item according to your level of knowledge or interest (1 star = low, 5 stars = high)

(IE1) Software knowledge (general)	☆ ☆ ☆ ☆ ☆
(IE2) Basic knowledge of how AI works	☆ ☆ ☆ ☆ ☆
(IE3) Experience with AI applications (private)	☆ ☆ ☆ ☆ ☆
(IE4) Experience with AI applications (professional)	☆ ☆ ☆ ☆ ☆
(IE5) Experience with data protection in the digital sector	☆ ☆ ☆ ☆ ☆
(IE6) Enjoy experimenting with new software	☆ ☆ ☆ ☆ ☆
(IE7) Interest in digital health technologies	☆ ☆ ☆ ☆ ☆
(IE8) Interest in using AI	☆ ☆ ☆ ☆ ☆

Private use of artificial intelligence

	daily	several times a week	once a week	less than once a week	never
(PU1) Text correction and improvement	○	○	○	○	○
(PU2) Text generation (content, emails, reports, etc.)	○	○	○	○	○
(PU3) Image generation / image processing	○	○	○	○	○
(PU4) Video generation and editing	○	○	○	○	○
(PU5) Speech-to-text transcription	○	○	○	○	○
(PU6) Translations	○	○	○	○	○
(PU7) Voice assistants	○	○	○	○	○
(PU8) Navigation and route planning	○	○	○	○	○

Artificial Intelligence in Medicine

	strongly disagree	disagree	neither agree nor disagree	agree	strongly agree
(UAT1) AI can be a useful tool to support medical diagnoses	○	○	○	○	○
(UAT2) The diagnostic performance of AI can be comparable tot hat of experienced specialists	○	○	○	○	○
(UAT3) AI can relieve physicians in everyday clinical practice and contribute to more efficient use of resources	○	○	○	○	○
(UAT4) The use of AI should be governed by ethical guidelines and data protection regulations	○	○	○	○	○
(UAT5) I trust AI recommendations similarly to the assessments of my colleagues	○	○	○	○	○
(UAT6) I critically evaluate AI suggestions before adopting them	○	○	○	○	○
(UAT7) I view AI as a supportice tool not as a replacement fo my clinicial decisions	○	○	○	○	○

Artificial intelligence in everyday clinical practice

For which tasks do you already use artificial intelligence (AI) in your daily clinical practice?

	daily	several times a week	once a week	less than once a week	never
(CP1) Image analysis	○	○	○	○	○
(CP2) Support in diagnosis	○	○	○	○	○
(CP3) Therapy recommendations	○	○	○	○	○
(CP4) Documentation / doctor's letters	○	○	○	○	○
(CP5) Communication with patients (e.g. chatbots, email writing)	○	○	○	○	○
(CP6) AI-powered translation tools	○	○	○	○	○
(CP7) Research activities	○	○	○	○	○
(CP8) Surgical simulators	○	○	○	○	○
(CP9) Exam preparation	○	○	○	○	○
(CP10) AI-based virtual case studies	○	○	○	○	○

AI use in diagnostics

	I already use AI for this	I would use AI for this	I believe AI is not appropriate for this purpose
(D1) Diabetic retinopathy	○	○	○
(D2) Age-related macular degeneration (AMD)	○	○	○
(D3) Glaucoma: Assessment of optic disc/OCT	○	○	○
(D4) Glaucoma: Visual field assessment	○	○	○
(D5) Corneal diseases (e.g. keratoconus)	○	○	○
(D6) Ocular tumors (e.g. retina, conjunctiva)	○	○	○
(D7) Uveitis	○	○	○
(D8) Fundus image analysis (general)	○	○	○
(D9) OCT image analysis (general)	○	○	○
(D10) Multimodal diagnostics	○	○	○

AI in medicine: decision-making, responsibility and ethical aspects

Ethical aspects & questions of responsibility

	strongly disagree	disagree	neither agree nor disagree	agree	strongly agree
(EL1) The data protection of sensitive patient data is not sufficiently clarified for me	○	○	○	○	○
(EL2) I still see unresolved liability and accountability issues related to AI misjudgments	○	○	○	○	○
(EL3) AI systems should be used exclusively as decision-support tools, not as autonomous decision-makers	○	○	○	○	○
(EL4) Patients should be explicitly informed and must give consent when their data are used for AI applications	○	○	○	○	○

Future expectations and training needs

*How do you assess the **future** role of AI in ophthalmology – and what training needs do you see?*

	strongly disagree	disagree	neither agree nor disagree	agree	strongly agree
(LN1) AI will be a central component of clinical decision-making in the coming years	○	○	○	○	○
(LN2) AI will improve the quality of patient care	○	○	○	○	○
(LN3) The use of AI will reduce the workload of doctors in the long term	○	○	○	○	○
(LN4) I would like to see structured training programs on the clinical application of AI	○	○	○	○	○
(LN5) I would use AI more often if I were better trained	○	○	○	○	○
(LN6) My current training prepares me sufficiently for the use of AI	○	○	○	○	○

Final assessment

In which areas of ophthalmology do you consider the use of AI to be particularly useful?
Min number of answers: 1   Max number of answers: 3

- ☐ Image analysis (e.g. OCT, fundus images)
- ☐ Screening & early detection (diabetic retinopathy, glaucoma, AMD)
- ☐ Automated documentation of findings and doctor's letters
- ☐ Triaging of referrals / emergencies (urgency classification)
- ☐ Treatment and course prognoses (e.g. progression in AMD and glaucoma)
- ☐ Intraoperative assistance (robotics)
- ☐ Surgical simulators
- ☐ Patient communication (chatbots, emails)
- ☐ Workflow optimization (scheduling, resource allocation)
- ☐ Tele-ophthalmology (remote evaluation of image data / home monitoring)
- ☐ Research / Scientific work
- ☐ additional use cases (optional comment): _______________

What are your main concerns about using AI in ophthalmology?
Min number of answers: 1   Max number of answers: 3

- ☐ Insufficient knowledge / lack of training
- ☐ Insufficient technological resources
- ☐ Legal, liability and data protection issues
- ☐ Lack of validated quality standards/guidelines
- ☐ Distrust in the reliability/accuracy of AI
- ☐ High implementation and licensing costs
- ☐ Bias and lack of diversity in training data
- ☐ Patient skepticism about the use of AI
- ☐ Lack of time in everyday clinical practice
- ☐ Complex operability / lack of user-friendliness of AI systems
- ☐ additional barriers (optional comment): _______________

Additional comments (optional)

Do you have any further comments, experiences, or suggestions regarding the use of AI in ophthalmology that have not yet been addressed in this survey? (Optional free-text response)

____
